# Quality monitoring of Shenmai injection by HPLC pharmacodynamic fingerprinting

**DOI:** 10.1186/s13065-023-00920-7

**Published:** 2023-03-25

**Authors:** Geng Yang, Shuai Li, Xiaoyi Sun, Yuanyuan Lv, Hongxia Huang

**Affiliations:** 1 School of Medicine, Hangzhou City University, Hangzhou, 310015 China; 2 Hangzhou TCM Hospital Affiliated to Zhejiang Chinese Medical University, Hangzhou, 310007 China

**Keywords:** Pharmacodynamic fingerprint, Shenmai injection, Principal component analysis, Multiple linear regression, Similarity analysis, Quality evaluation

## Abstract

A probable problem of disconnection between chemical fingerprints and drug effects for TCMs would be contrary to the original intention of fingerprint research, and limits the development and application of fingerprints. In this study, Shenmai injection, as a treatment dosage form of coronary heart disease, shock, and viral myocarditis clinically, was applied as the research object. The fingerprint of Shenmai injection was constructed, and the pharmacodynamic test of antioxidant effect was carried out to obtain quantitative characteristics and pharmacodynamic data. On this basis, a monitoring model based on the HPLC pharmacodynamic fingerprint was established to evaluate the quality of Shenmai injections from different batches and different manufacturers. Results showed that the optimized HPLC method had good repeatability, precision, and stability. A total of 28 characteristic peaks were identified to provide more chemical information. Furthermore, 13 ginsenosides and notoginsenoside have been selected as characteristic components of LC/MS fingerprint method. 8 peaks closely related to antioxidant properties by multiple linear regression method, which were identified as Rg1, Re, Rf, Rb1, and some other ginsenosides using MS analysis. The monitoring model based on HPLC pharmacodynamic fingerprint could successfully identify quality differences for Shenmai injections. Based on the case study of Shenmai injection, the novel and practical fingerprint analytical strategy could be further applied to monitor or predict the quality of TCMs.

**Supplementary Information:**

The online version contains supplementary material available at 10.1186/s13065-023-00920-7.

## Introduction

Traditional Chinese medicine (TCM) has played a vital role in disease prevention and treatment in China for thousands of years. TCM injections as a new pharmaceutical form in the modernization of TCM, are also widely applied in clinical work [1, 2]. However, an increasing number of adverse drug reactions (ADRs) of TCM injections has been reported [3–5], which hinder their broader application and popularity among international healthcare practitioners. Therefore, establishing a rapid and practical quality evaluation method according for TCM injections to the existing problems would be critical for the development of TCM industry [6, 7].

The TCM fingerprint atlas refers to a chromatogram that can mark the characteristics of TCMs has been widely accepted nationally and internationally [8–11]. The China Food and Drug Administration (CFDA) has officially required all the TCM injections to be standardized by chromatographic fingerprints since 2000 [12]. In addition, this chemical fingerprint method is specified for quality control of herbal medicine by many authorities, including the United State Food and Drug Administration (FDA) and European Medicines Evaluation Agency (EMEA) [13, 14]. However, there is a probable problem of disconnection between chemical fingerprints and drug effects. Attention should also be paid on pharmacodynamics research for comprehensively evaluating TCMs quality [15]. To date, studies focused on chemical fingerprint of TCMs were abundant, while a few studies were reported on the connection between chemical fingerprints and drug effects.

Pharmacodynamic fingerprint refers to “chemical fingerprints are related to their efficacy” analysis [16–18], which can make up for a common defect in the current research on chemical fingerprints of TCMs. There are three stages to establish a pharmacodynamic fingerprint of TCM [19]. First is chemical fingerprint acquisition stage. Image or spectrum of component groups that can characterize the chemical composition of the sample is captured by spectroscopic, chromatographic, and coupled techniques for instrumental analysis. The second stage is establishing a suitable pharmacodynamic model to obtain biological fingerprints and multiple effect-related components. The third stage is developing pharmacodynamic fingerprint applying mathematical methods to correlate chemical fingerprints and pharmacological activity data [18]. Therefore, how to obtain representative chemical fingerprints, what pharmacodynamic model or index to use to characterize the efficacy of the drug, and what kind of analysis method is used to assess the overall similarity quickly and simply between different sample fingerprints, are the core link of fingerprint evaluation system.

Shenmai injection as a research object has the largest sales among TCMs in China. There are 66 registration certificates for Shenmai Injection according to the CFDA database. It is prepared from *red ginseng* and *Radix Ophiopogonis*, and is considered to invigorate *Qi* to relieve desertion, nourish *Yin*, and promote saliva production in Chinese medicine. Clinically, this injection is commonly used in treatment of coronary heart disease, shock, and viral myocarditis [20–22]. Convincing clinical experiences also showed Shenmai injection as supplemental treatments has exhibited remarkable efficacy in treatment and rehabilitation of liver cancer and COVID-19 [23–25]. There are some studies found Shenmai injection had antioxidant effect, which could decrease oxidative stress [26]. Xiao WH et al. [27] used FRAP, DPPH·, NO·, H_2_0_2_· free radical scavenging methods to test the antioxidant capacity of Shenmai injections. The results show that Shenmai injection has a certain invitro antioxidant capacity, and the activity is concentration-dependent. A recent study also reported that Shenmai injection has antioxidant effects by reducing ROS and eliminating the production of oxidizing effect such as malondialdehyde (MDA), which may decrease the cardiovascular oxidative stress induced by Nitroglycerin (NTG) tolerance [28–30]. The mechanism was revealed to be by modulating the Nrf2/Keap1 signal pathway, resisting oxidative stress injury [31]. The efficacy of Shenmai injection is closely related to the antioxidant effect according to the previous reports. The main substance in Shenmai injection is ginsenoside (such as Rg1、Re、Rf、Rg2、Rb1、Rd、Rc), ophiopogonin D, Fructose, et al. [32–34]. And ginsenosides have been identified as the most important active ingredient. According to the National Drug Standard (WS3-B-3428-98-2010Z) issued by CFDA [35], the similarity of the 16 characteristic peaks of the chromatographic fingerprint should not be less than 0.85 to ensure standardization. In addition, the content of ginsenosides is specified. However, the separation efficiency of this method is low [36]. To date, only 16 characteristic peaks have been reported, as well as the lack of identification of the characteristic peak components and pharmacodynamic evaluation.

In this study, high-performance liquid chromatography (HPLC) was used to establish a chemical fingerprint for Shenmai injection. Crystal violet method [37, 38] was applied to determine the intensity of antioxidant effect. And the HPLC pharmacodynamic fingerprint of Shenmai injection combined the chemical fingerprint and antioxidant activity data by multivariate statistical analysis was established to evaluate the quality of injections from different manufacturers in different batches. The main components for the characteristic peaks in the fingerprint of the Shenmai injection were identified using mass spectrometry (MS). This research provides technical support for quality control of Shenmai injection and other TCMs.

## Experiment and methods

### Chemicals and instruments

#### Reagents

Acetonitrile and methanol are all gradient grade (Merck, Germany). Glacial acetic acid is purchased from Yonghua Chemical Technology Co., Ltd. (HPLC grade, China). Purified water is supplied by Milli-Q Reference. Phosphate buffer is purchased from Shandong Linyi Yong'an Laboratory (pH 7.0, 0.5 mol L^−1^, China). It was filtered through a membrane filter with a pore size of 0.22 μm. Crystal violet is purchased from Shanghai Qiangshun Chemical Reagent Co., Ltd (Batch No. 20140422, China). Potassium hydrogen phthalate, 30% hydrogen peroxide, and ferrous sulfate heptahydrate are all purchased from Jiangsu Yonghua Chemical Technology Co., Ltd (Batch No. 20140410, 20140815, and 20140630 respectively, China).

#### Samples and sample preparation

Thirty batches of Shenmai injection were obtained from three manufacturers, which are represented by the letters A, B, and C. Seventeen batches were collected from Manufacturer A, six batches from Manufacturer B, and seven batches from Manufacturer C. Before HPLC and MS analysis, each sample was centrifuged at 10,000 rpm for 10 min and the supernatant was retained.

#### Instruments

The HPLC (1220 Infinity, Agilent Technologies) equipped with a quaternary gradient pump, online degasser, autosampler, column oven, ultraviolet detector, and ChemStation software were used. The chromatographic column was a ZORBAX SB-C_18_ (250 mm × 4.6 mm i.d., 5.0 µm particle size; Agilent Technologies). For HPLC/MS, we used a 5600 + quadrupole time-of-flight MS (Sciex). We also used a Vis- spectrophotometer (Spectrumlab 22pc, Shanghai Lengguang Technology Co., Ltd), centrifuge (Eppendorf Minispin, Sigma-Aldrich), ultrasonicator (USC702, Shanghai Bolong Electronic Equipment Co., Ltd), and water purifier (Milli-Q Reference, Millipore Sigma).

### Methods

#### Chromatographic conditions

The HPLC analysis was conducted using the conditions described for Shenmai injection in The National Drug Standards from the CFDA (WS3-B-3428-98-2010Z) [35]. The mobile phase composition, column temperature, and mobile phase gradient were optimized. HPLC/MS separation were used the same column and optimized gradient.

#### Crystal violet spectrophotometric method [38]

The basic principle of the clearance rate ·OH of crystal violet spectrophotometric method is as follows:

1 $$ {\text{Fe}}^{{{2} + }} + {\text{H}}_{{2}} {\text{O}}_{{2}} \to {\text{Fe}}^{{{3} + }} + \cdot {\text{OH }} + {\text{OH}}^{ - } $$

·OH attacks the point of high electron cloud density, and reacts electrophilically with the C=C group in crystal violet to cause crystal violet to fade. ·OH generation the amount can be indirectly measured by capturing the change rate of crystal violet absorbance value. Theoretically, antioxidants can scavenge hydroxyl radicals and increase the absorbance of the system.

1 mL of 1.0 mmol L^−1^ FeSO_4_ solution, 0.75 mL of 0.4 mmol L^−1^ crystal violet, 0.1 mL potassium hydrogen phthalate buffer (PH = 4), and 0.7 mL of a certain concentration of H_2_O_2_ solution were added in a 25 mL volumetric flask, dilute to 25 mL with water, then shake well. The absorbance A_b_ was measured at a certain wavelength after standing for a while, and meanwhile the absorbance A_0_ at a certain wavelength with no H_2_O_2_ solution is measured. The hydroxyl radicals would be generated. The amount is characterized by.

△A = A_0_ − A_b_. If the absorbance A_S_ was measured after a certain volume of Shenmai injection was added, the hydroxyl radical scavenging rate CLR% could be calculated according to Eq. 2.

2 $$ {\text{CLR}}\% \, = \, \left( {{\text{A}}_{{\text{S}}} - {\text{A}}_{{\text{b}}} } \right)/\left( {{\text{A}}_{0} - {\text{A}}_{{\text{b}}} } \right) \, \times { 1}00\% $$

#### MS conditions

MS was conducted in both positive and negative ion modes. The scan range was m/z 100–2000. In positive ion mode, the ion source voltage was 5.5 kV, and the ion source temperature was 600 °C. In negative ion mode, the ion source voltage was − 4.5 kV, and the ion source temperature was 550 °C. The gas 1 (air) and gas 2 (air) pressures were set at 50 psi. The curtain gas (nitrogen) pressure was set at 35 psi. The maximum allowable error was ± 5 ppm. The dispersive potential was 100 V and the collision energy was 10 V.

#### Principal component analysis

PCA is a common multivariate statistical method that reduces the dimensionality to simplify data sets and reduce the number of indices. It is a common pattern recognition method, and is especially useful for evaluating chemical fingerprints. In the present study, the peak area of each identified peak was considered as one dimension in the data set. PCA involves orthogonal transformation of original random variables into new variables that are not related to the original observations [39]. According to the feature extraction point of view, PCA is equivalent to an extraction method that is based on minimum mean square error.

The area and retention time data from the characteristic peaks for the 30 samples were standardized, and PCA was performed using the chemometric software Unscrambler 9.7 (CAMO Software Inc, Woodbridge).

#### Multiple linear regression

Multiple linear regression attempts to model the relationship between two or more explanatory variables* x*_*j*_ (*j* = 1, 2, 3, …, n) and a response variable *y* by fitting a linear equation [40]. Every value of the independent variable *x* is associated with a value of the dependent variable *y*.

3 $$y={b}_{0}+{b}_{1}{x}_{1}+{b}_{2}{x}_{2}+\dots +{b}_{j}{x}_{j}+e$$

In the Eq. 3, *b*_*0*_ is the regression constant; *b*_*j*_ (*j* = 1, 2, 3, …, n) is the regression parameter; *e* is the random error.

#### Similarity evaluation

The similarity method uses a certain number of common and featured indices in two samples as a unified measure [16]. Decision principles are used to describe the degree of matching between these two samples. Compared with chemical pattern recognition, the similarity method focuses more on the mutual influence and intrinsic correlation among vector elements. Methods include the distance coefficient, similarity coefficient, relative correlation degree, improved *Nei* coefficient, and support vector machine [41, 42], and the included angle cosine method is the most common similarity evaluation method [43]. In this paper, fingerprint similarity was evaluated using a similarity evaluation system published by the Chinese Pharmacopoeia Commission. Based on the included angle cosine method, this system uses the basic equation for the vector angle cosine to calculate the similarity between two fingerprints from Eq. 4.

4 $$Similarity (\overrightarrow{X}, \overrightarrow{Y})= cos (\theta ) =\frac{{\sum }_{\mathrm{i}=1}^{\mathrm{n}}{\mathrm{x}}_{\mathrm{i}}{\mathrm{y}}_{\mathrm{i}}}{{\sum }_{\mathrm{i}=1}^{\mathrm{n}}{{\mathrm{x}}_{\mathrm{i}}}^{2}{\sum }_{\mathrm{i}=1}^{\mathrm{n}}{{\mathrm{y}}_{\mathrm{i}}}^{2}}$$

where *x*_*i*_ and *y*_*i*_ are components of vector $\overrightarrow{X}$ and $\overrightarrow{Y}$ respectively. The resulting similarity close to 1 meaning exactly the same, and with 0 indicating decorrelation.

## Results and discussion

### Optimization of the HPLC conditions

#### Mobile phase composition

Methanol–water, methanol-acetic acid–water, methanol-phosphate buffer-water, acetonitrile–water, and acetonitrile–phosphate buffer-water were compared as mobile phase systems. Among these systems, the separation of acetonitrile–phosphate buffer-water is slightly better than the acetonitrile–water system, but it was not easy to maintain the instrument (especially for column and pump) and had a poor repeatability. The mobile phase system containing methanol (including methanol–water, methanol-acetic acid–water, and methanol-phosphate buffer-water) was inferior to mobile phase systems containing acetonitrile. Finally, acetonitrile–water was selected as the mobile phase.

#### Column temperature

With acetonitrile–water as the mobile phase, and 203 nm as the detection wavelength, we investigated the method performance with column temperatures of 25 °C, 30 °C, and 40 °C. The column temperature had a relatively small effect on the fingerprint of the Shenmai injection, and there was only a slight difference in the retention time with changes in the temperature. Therefore, a column temperature of 25 °C (i.e. room temperature) was selected for HPLC.

#### Mobile phase gradient

Chromatographic separation is greatly affected by the mobile phase gradient, and optimization of the mobile phase gradient conditions enables good separation of major peaks. In the presents study, using the optimized mobile phase composition and column temperature, we optimized the mobile phase gradient. The optimum mobile phase gradient elution procedure is detailed in Table 1.

**Table 1:** Gradient elution conditions for HPLC fingerprinting of Shenmai injection

Time (min)	Solution A: acetonitrile (%)	Solution B: water (%)
0	0	100
20	5	95
30	20	80
45	28	72
75	60	40
80	90	10
90	90	10

**Fig. 1:**
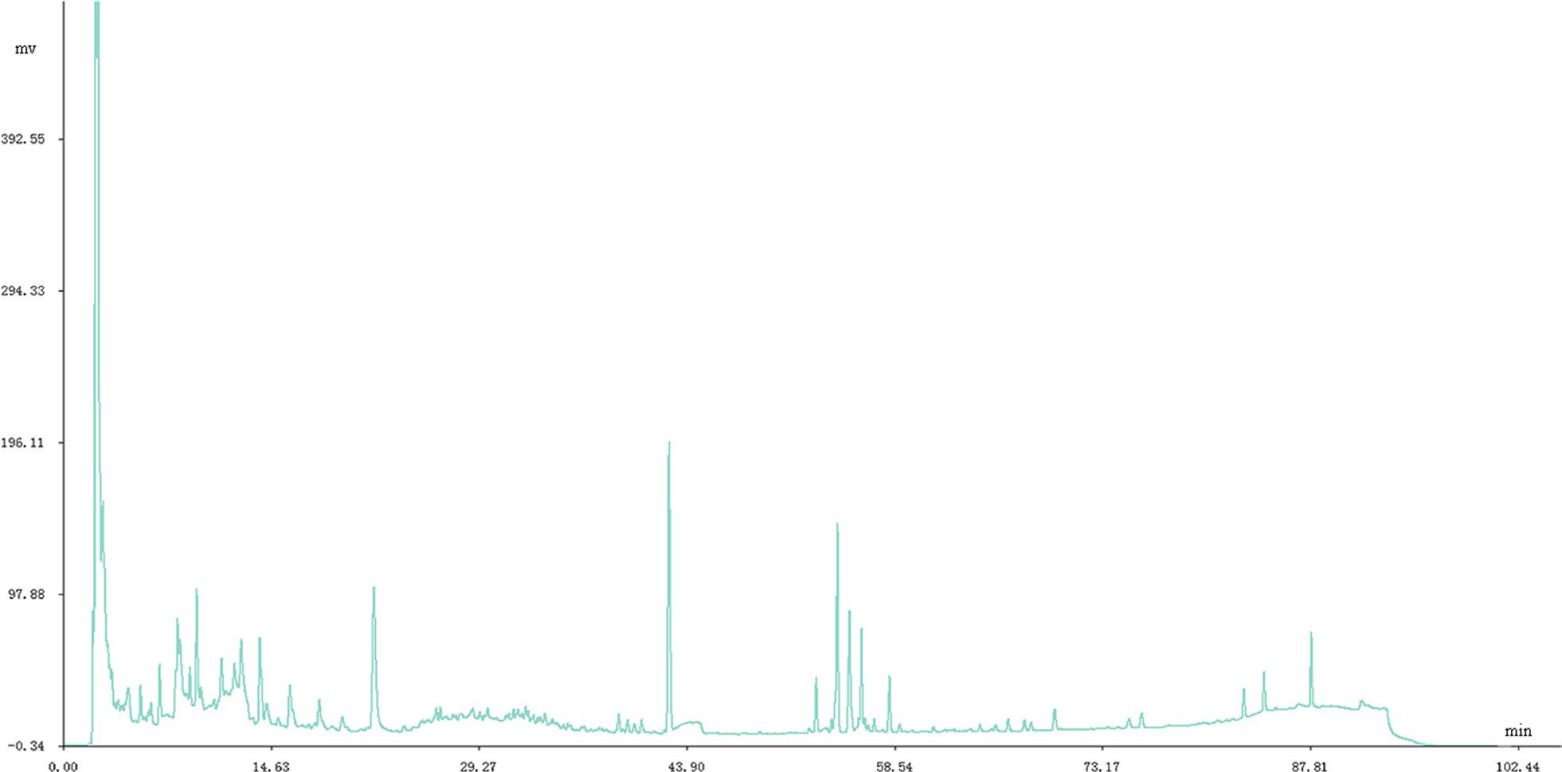
HPLC fingerprint of Shenmai injection

In summary, the optimum HPLC conditions for analysis of the Shenmai injection were as follows: detection wavelength of 203 nm, column temperature of 25 °C, chromatographic column of ZORBAX SB-C_18_ (4.6 mm × 250 mm, 5.0 µm; Agilent Technologies), mobile phase of acetonitrile–water, injection volume of 20 µL, and the mobile phase gradient shown in Table 1. Under these conditions, the sample was analyzed to obtain a HPLC fingerprint of Shenmai injection (Fig. 1).

### Establishment of the HPLC fingerprint for shenmai injection and method validation

**Fig. 2:**
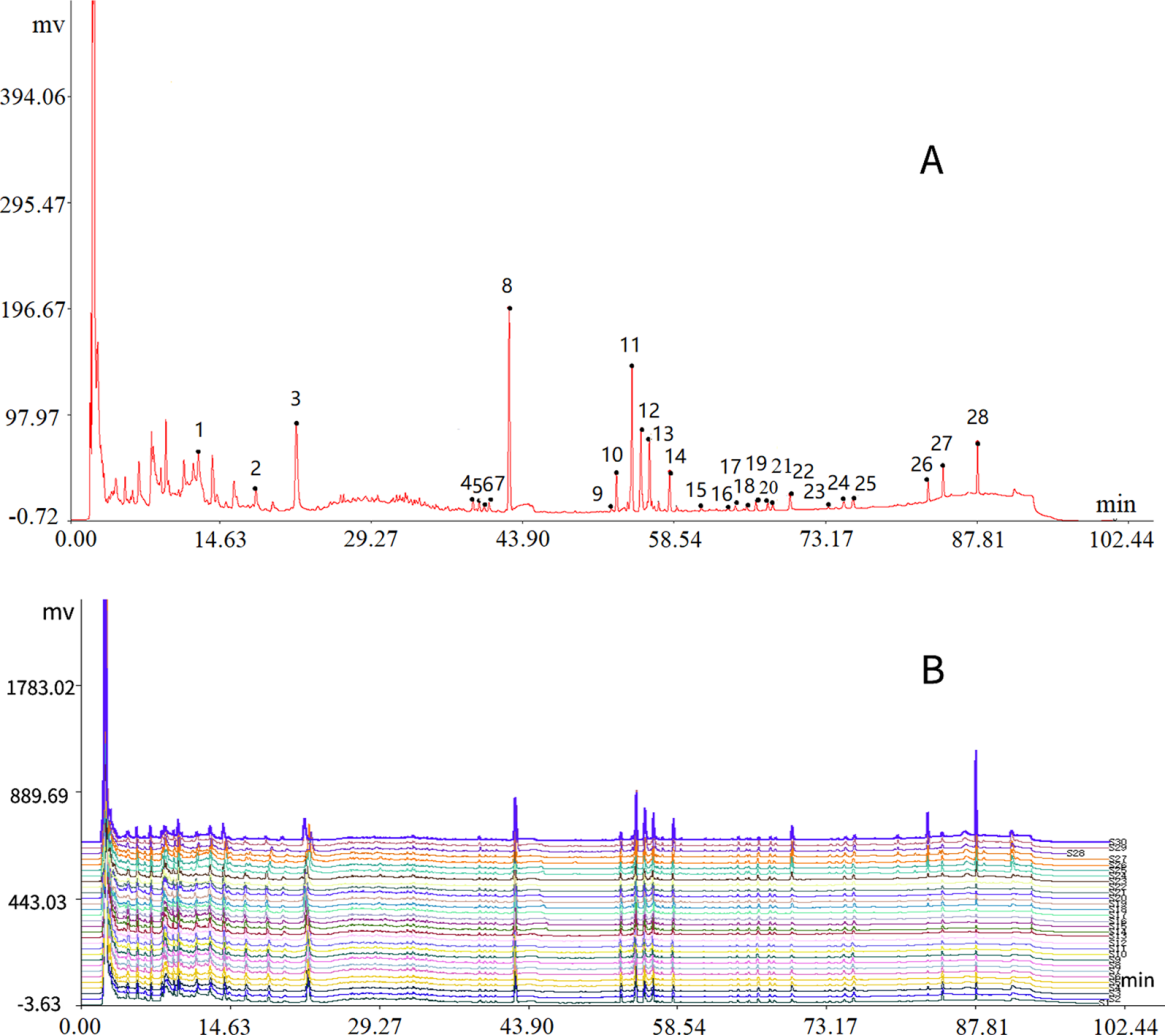
HPLC fingerprint of Shenmai injection and its main chromatographic peaks. **A** A representative HPLC fingerprint; **B** All 30 sample overlapped HPLC fingerprints

A chromatographic fingerprint for Shenmai injection was established by determining what peaks were characteristic. Among the chromatographic peaks detected for the 30 batches of Shenmai injection from the three manufacturers, 28 peaks were detected in all of the samples. These peaks showed good separation and high peak intensities, and could be used as fingerprint peaks for Shenmai injection (Fig. 2). Combined, these 28 peaks accounted for more than 90% of the total peak area.

#### Precision

We injected sample A_1_ 6 times using the optimized conditions detailed in “Chemicals and Instruments”, and recorded and analyzed the peak areas and retention times of the 28 characteristic peaks. The retention time RSDs of the 28 characteristic peaks were all less than 1%, and the peak area RSDs were less than 5%, except for peaks 2 and 27 (Additional file 1: Table S1). The areas of peaks 2 and 27 were less than 10% of the total area, which means they do not have a large contribution to the overall precision. Therefore, the chromatographic fingerprinting method has a good precision.

#### Repeatability

Six samples from the same batch from Manufacturer A were used to investigate the repeatability. The RSDs for the peak areas and retention times of the 28 characteristic peaks were calculated. The retention time RSDs for the 28 characteristic peaks were all less than 1%, and the peak area RSDs were all less than 5%, except for those for peaks 2, 9, 16, 17, 18, and 27 (Additional file 1: Table S2). Combined, these 6 peaks contributed less than 10% of the total peak area. They do not account for a large proportion of the peak area, which means they do not have a large contribution to the overall repeatability. Therefore, this method has good repeatability.

#### Stability

One sample from Manufacturer A was analyzed at 0, 4, 8, 12, 16, 20, and 24 h. The retention time RSDs of the 28 characteristic peaks were all less than 1%, and the peak area RSDs were all less than 5%, except for those for peaks 2, 9, and 24 (Additional file 1: Table S3). These 3 peaks contributed less than 5% of the total peak area, which means they do not have a large contribution to the overall stability. Therefore, the Shenmai injection is relatively stable for up to 24 h.

In summary, the HPLC fingerprint obtained from the Shenmai injection sample under the optimized conditions (“Chemicals and Instruments”) had 28 characteristic peaks, which is far more than the 16 characteristic peaks in the relevant standard (WS3-B-3428-98-2010Z) [35].

Therefore, the chromatographic fingerprint established in this paper contains more features for identification than the current standard. In addition, the developed method has good precision, stability, repeatability, and reproducibility.

### Antioxidant determination

Through the screening of detection wavelength, reaction time, and H_2_O_2_ concentration, the optimal conditions were determined for the antioxidant experiment as follows: detection wavelength was 590 nm, reaction time was 40 min, and H_2_O_2_ solution concentration was 0.005%. The RSDs of precision, stability, repeatability, and reproducibility of Antioxidant determination method were all below 1.0%.

According to the final optimized antioxidant test conditions, a total of 30 batches of Shenmai injection from 3 manufacturers were tested for free radical scavenging rate, and the antioxidant properties of the samples were obtained. The results are shown in Table 2.

**Table 2:** Results of free radical scavenging rate of 30 batches of Shenmai injection

Batch No	CLR%	Batch no	CLR%	Batch no	CLR%
A_1_	92.31	A_11_	93.40	B_4_	97.43
A_2_	97.89	A_12_	94.34	B_5_	96.12
A_3_	93.20	A_13_	94.58	B_6_	96.79
A_4_	97.89	A_14_	95.20	C_1_	99.30
A_5_	90.74	A_15_	95.00	C_2_	88.44
A_6_	94.37	A_16_	97.57	C_3_	98.46
A_7_	96.20	A_17_	98.45	C_4_	98.70
A_8_	92.56	B_1_	96.48	C_5_	96.34
A_9_	94.30	B_2_	95.77	C_6_	98.15
A_10_	91.98	B_3_	92.50	C_7_	94.27

Table 2 shows that the free radical scavenging rates of 30 batches of Shenmai injection are all above 90%, which indicating injections have better antioxidant properties, except for sample C_2_.

### Chromatographic-pharmacodynamic relationship

Taking the peak area of the 28 common peaks in the HPLC fingerprint of 30 batches of Shenmai injection as the independent variable (*x*), and the dependent variable is the antioxidant value (*y*), Using PASW Statistics 18 statistical analysis software to establish multiple regression linear equations. Filter out 8 variables, namely $x_{2} , \, x_{3} , \, x_{4} , \, x_{5} , \, x_{6} , \, x_{9} , \, x_{11} , \, x_{13}$ to get the linear equation:

$$ y =\,114.559 + 0.017x_{3} - 0.062x_{4} + 0.017x_{8} - 0.080x_{10} + 0.024x_{11} - 0.042x_{12} + 0.284x_{13} + 0.026x_{14} \,({\text{P}}\, < \,0.0{5},{\text{ R}}^{{2}} \, = \,{1}.000) $$

### MS results for the characteristic peaks

**Fig. 3:**
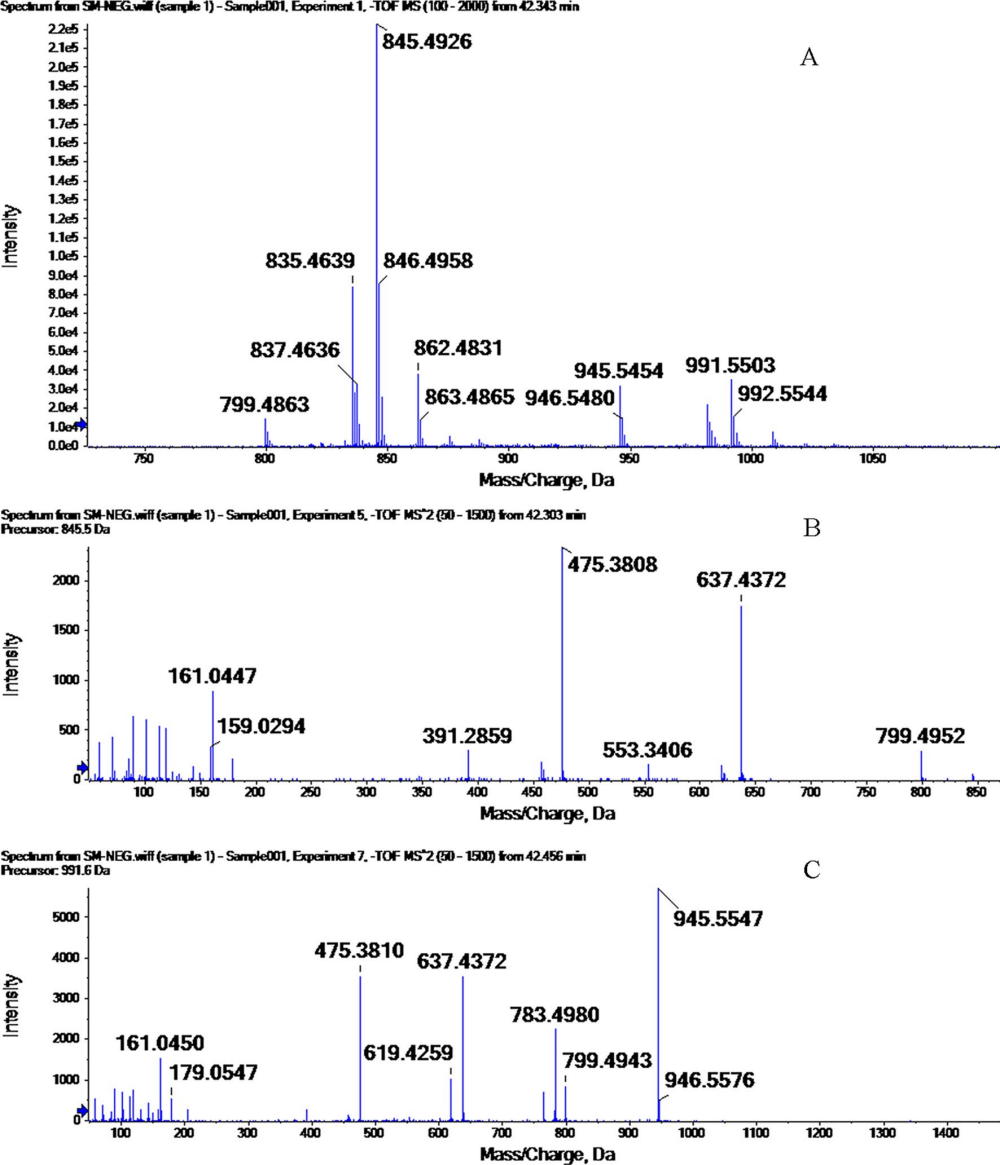
The MS spectra of peak 8 of Shenmai injection. **A** First order mass spectrum; **B** Second order mass spectrum of the precursor ion at m/z 845; **C** Second order mass spectrum of the precursor ion at m/z 991

The 28 characteristic peaks in the chemical fingerprint of a batch of Shenmai injection were analyzed by HPLC/MS. According to the 3.4, the 8 molecular ions as well as other characteristic peaks, main fragment ions and proposed formulae of the compounds are summarized in Table 3. The characteristic peaks were identified based on their LC–MS results, and by comparison with the literature [44–46]. The main active components of Shenmai injection, such as Rg1, Re, Rf, Rb1, and some other ginsenosides were identified among the HPLC fingerprint peaks. For example, the first and second order mass spectra of peak 8 are shown in Fig. 3. The first order mass spectrum showed a strong signal at m/z 845, which was attributed to the molecular ion peak with a formic acid (m/z 799). The product ion spectrum of the m/z 799 precursor ion had peaks at m/z 637 and 475, along with other characteristic peaks. The fragment ion at m/z 475 was attributed to a ginseng triol-type saponin, and that at m/z 637 was formed by loss of a sugar chain from the m/z 799 compound. Signals at m/z 945 and 991 were stronger in the first order spectrum than the second order spectrum. The peak at m/z 991 was attributed to formic acid adduct ions (m/z 945). The product ion spectrum of the m/z 945 precursor ion contained characteristic peaks at m/z 799, 637, and 475. These were formed by removal of 162 sugar chains in succession from the precursor. The fragment ion at m/z 637 produced from the parent ion at m/z 945, was attributed to the loss of a disaccharide consisting of a deoxyhexose and a hexose, which indicated that the hexose in the disaccharide was directly attached to the saponin aglycone. Similarly, the fragment ion at m/z 475 was produced by loss of all three sugar units from the parent ion at m/z 945. Finally, two components were identified as Rg1 and Re in comparison with the retention time and mass spectral data with those of the standard, and the reported literature [45, 46].

**Table 3:** LC/MS analysis of main constituents of Shenmai injection fingerprint

Peak	Identification	[M-H]^−^(m/z)	Fragment ion (m/z)
3	NotoginsenosideR_1_	931.5	799.5,637.4[M-H-Glc]^−^; 637.4[M-H- Glc-Xyl]^−^;475.4[M-H-Glc-Xyl-Glc]^−^
4	20-Glc-ginsenoside-Rf	961.5	799.7[M-H-Glc]^−^;653.3[M-H-2Glc]^−^;475.4[M-H-3Glc]^−^
5,6,7	NotoginsenosideR_2_	769.5	637.4[M-H-Xyl]^−^;475.4[M-H-Xyl-Glc]^−^
8	Ginsenoside-Re	945.5	783.5[M-H-Rha]^−^;637.4[M-H-Rha-Glc]^−^;475.4[M-H-Rha-2Glc]^−^
8	Gnsenoside-Rg_1_	799.5	637.4[M-H-Glc]^−^;475.4[M-H-2Glc]^−^
9	Ginsenoside-Rf	799.5	637.4[M-H-Glc]^−^;475.4[M-H-2Glc]^−^
10	Ginsenoside-Ra_1_/Ra_2_	1209.6	1047.5[M-H-Glc]^−^;901.4[M-H-Glc-Xyl]^−^;759.4[M-H-Glc-Xyl-Araf]^−^
11	Ginsenoside-Rb_1_	1107.6	945.5[M-H-Glc]^−^;783.5[M-H-2Glc]^−^;621.4[M-H-3Glc]^−^;459.6 [M-H-4Glc]^−^
12	Ginsenoside-Rb_2_	1078.6	945.5[M-H-Glc]^−^;783.5[M-H-2Glc]^−^; 621.4[M-H-3Glc]^−^;459.4 [M-H-3Glc-Arap]^−^
13	Ginsenoside-Rb_3_	1078.6	945.5[M-H-Glc]^−^;783.5[M-H-2Glc]^−^; 621.4[M-H-3Glc]^−^; 459.4 [M-H-3Glc-Xyl]^−^
14	Ginsenoside-Rd	946.0	783.5[M-H-Glc]^−^;621.5[M-H-2Glc]^−^;459.6 [M-H-4Glc]^−^
15	Ginsenoside-Rg_3_	783.5	637.4 [M-H-Glc]^−^;475.4[M-H-2Glc]^−^
16	Ginsenoside-Rg_6_/F_4_	765.5	603.4 [M-H-Rha-H_2_O]^−^;457.5[M-H -Rha-Glc]^−^
17,18,19	Ginsenoside-Rh_4_	620.8	619.8 [M-H-Rha]^−^; 601.5[M-HRha-H_2_O];457.5[M-H -Rha-Glc]^−^
20,21	20(R)-ginsenoside-Rg_3_	783.5	621.4[M-H-Glc]^−^;459.4[M-H-2Glc]^−^
24,25	20-Glc-ginsenoside-Rf	765.3	603.3[M-H-Glc]^−^

*Glc* β-D-glucose, *Xyl* β-D-xylose, *Rha* α-L-rhammose, *Arap* α-L-arabinose(pyranose), *Araf* α-L-arabinose(furanose)

In the MS results, the two main components of peak 8 were not completely separated. This occurred because the retention behaviors of Rg1 and Re are similar, and the chromatographic conditions should be optimized for this in future. Reports of complete separation of Rg1 and Re are rare in the literatures [44, 45]. A few studies [47, 48] optimized the gradient elution to solve this problem, but this resulted in a lengthy analysis.

Because of the complexity of the injection process (preparation of the actual Shenmai injection including extraction, concentration, purification, et al.), the MS results for some components were affected by components from the matrix, such as plasticizers. Consequently, the spectra lacked characteristic fragments or contained new components produced by chemical reactions that occurred during processing. Because of a lack of relevant data in the literature, the structures of seven characteristic peaks (peak 1, 2, 22, 23, 26, 27, 28) in the spectra were unable to be identified. These unidentified components were thought to form from ginsenoside diol and triol saponins, but further experimental analysis is required to confirm this.

### Establishment and application of a quality control model for shenmai injection

#### PCA analysis

**Fig. 4:**
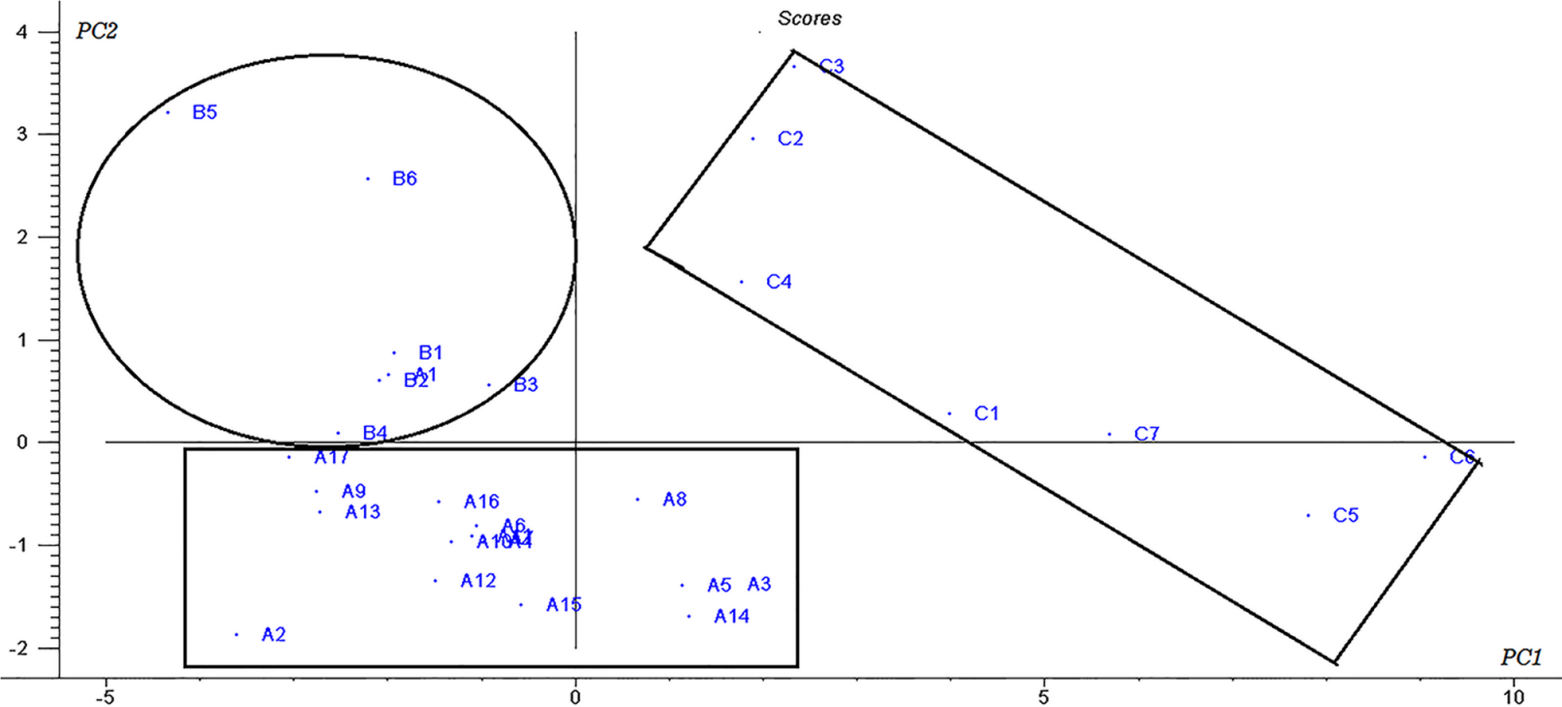
PCA analysis score plot of Shenmai injection

Fingerprint data from the 30 batches of Shenmai injection were standardized and then imported into Unscrambler software (Version 9.7) for PCA analysis. Two principal components, PC_1_ and PC_2_, explained 92% of the variance. The distributions of Shenmai injections from the three manufacturers were significantly different in the score plot (Fig. 4). The quality differences between the batches from Manufacturer A were small (i.e., the sample distribution was relatively concentrated). Quality differences between samples from manufacturer C and those from manufacturers A and B were obvious, and between-batch quality differences were large. This method is visual and intuitive and can be used to distinguish Shenmai injections from different manufacturers and different batches.

#### Similarity analysis

The fingerprint data for the 30 batches of Shenmai injection from three manufacturers were imported into a similarity evaluation system published by the Chinese Pharmacopoeia Commission.

First, the average fingerprints of the Shenmai injections for each manufacturer were used as standard spectra to calculate the similarity among different batches of Shenmai injection from the same manufacturer (Table 4). The obtained similarity (*R*) was greater than 95%, indicating that between-batch quality differences were small and the quality was stable and uniform.

**Table 4:** Results of similarity evaluations between batches from a single manufacturer

Batch No	R%	Batch no	R%	Batch no	R%
A_1_	98.3	A_11_	97.7	B_4_	96.0
A_2_	98.9	A_12_	96.3	B_5_	96.7
A_3_	95.9	A_13_	97.4	B_6_	97.3
A_4_	98.8	A_14_	97.5	C_1_	97.6
A_5_	96.1	A_15_	97.0	C_2_	96.8
A_6_	98.7	A_16_	97.7	C_3_	97.3
A_7_	98.5	A_17_	98.0	C_4_	98.0
A_8_	97.9	B_1_	97.3	C_5_	98.0
A_9_	98.9	B_2_	98.6	C_6_	97.8
A_10_	97.4	B_3_	96.8	C_7_	97.9

Next, the spectra from all 30 batches of samples were averaged to produce a standard fingerprint and calculate the similarity of the fingerprints of Shenmai injection (Table 5). The similarity of the Shenmai injection spectra between different manufacturers was greater than 90% for all samples, except for sample C_2_. It indicated that indicating that different manufacturers’ quality differences were also small. Observing the specific similarity value, the Shenmai injections produced by manufacturers A and B were similar (no statistical difference in similarity), whereas the spectral similarity of the Shenmai injections produced by Manufacturer C was relatively poor. This result is consistent with the PCA analysis results.

**Table 5:** Results for evaluation of sample similarity between batches

Batch No	R%	Batch no	R%	Batch no	R%
A_1_	100.0	A_11_	96.1	B_4_	94.5
A_2_	95.9	A_12_	93.5	B_5_	95.5
A_3_	93.3	A_13_	93.8	B_6_	95.1
A_4_	95.9	A_14_	93.1	C_1_	93.7
A_5_	92.1	A_15_	93.9	C_2_	89.0
A_6_	95.8	A_16_	94.7	C_3_	90.9
A_7_	94.8	A_17_	93.9	C_4_	93.1
A_8_	95.4	B_1_	96.5	C_5_	91.8
A_9_	95.3	B_2_	99.0	C_6_	91.8
A_10_	98.1	B_3_	95.3	C_7_	92.1

Similarity analysis by angle cosine in this paper was not sensitive to the change in the concentration of the constituents. When the peak area of fingerprint distribution was wide, the methods are not sensitive to the difference of data. Therefore, the similarity change caused by the change of the characteristic peaks content in the fingerprint could not be accurately evaluated.

In summary, results showed the good performance of the quality control framework for Shenmai injection. HPLC pharmacodynamic fingerprint of Shenmai injection enriched chemical and pharmacodynamic information for identification. The number of characteristic peaks was expanded from 16 to 28 compared with the standard method by optimizing the original HPLC method. Among them, 8 peaks identified as Rg1, Re, Rf, Rb1, and some other ginsenosides using MS analysis were closely related to antioxidant properties by MLR method. Furthermore, the monitoring model based on HPLC pharmacodynamic fingerprint could successfully identify differences in quality for Shenmai injections from different manufacturers and different batches.

## Conclusions

In this study, the HPLC pharmacodynamic fingerprint of Shenmai injection as a quality control strategy was constructed. The optimized HPLC method with good repeatability, precision, and stability provided 28 characteristic peaks, and the pharmacodynamic test of antioxidant effect was carried out to obtain quantitative characteristics and pharmacodynamic data. Among these characteristic peaks, 8 peaks identified as Rg1, Re, Rf, Rb1, and some other ginsenosides using MS analysis, were closely related to antioxidant properties by MLR method. The monitoring model based on HPLC pharmacodynamic fingerprint could successfully identify quality differences for Shenmai injections. The results provide technical support for the basic research on Shenmai injection.

Based on this study and correlative reports of the ginsenosides’ bio-activities, further pharmacology tests of the ginsenosides in Shenmai injecion would be designed and the indexes for pharmacodynamic fingerprint could be optimized to improve the quality control efficiency in future research. And the novel and practical HPLC pharmacodynamic fingerprint analytical strategy could be further applied to monitor or predict the quality of TCM injections, which can greatly facilitate the development of quality evaluation, enhance the clinical safety and effectiveness of TCMs.

## Supplementary Information

**Additional file 1.** The HPLC fingerprint evaluation of precision, repeatability, and Stability.

## Data Availability

All data generated in this study are included in this article and additional files. Material is available from the corresponding author on reasonable request.

## Abbreviations

TCM: Traditional Chinese medicine
CFDA: China Food and Drug Administration
LC–MS: Liquid chromatography- mass spectrometry
HPLC: High-performance liquid chromatography
PCA: Principal component analysis
