# Supplementary material for: Quality monitoring of Shenmai injection by HPLC pharmacodynamic fingerprinting
Source: BMC Chem. 2023 Mar 25;17(1):28. doi: 10.1186/s13065-023-00920-7 (PMC10039686; doi:10.1186/s13065-023-00920-7)
Supplement: Supplementary file 1 — Additional file 1. The HPLC fingerprint evaluation of precision, repeatability, and Stability. [file 13065_2023_920_MOESM1_ESM.docx]

**Supplementary material**

**Table S1.** Precision evaluation: relative retention times and relative peak areas

| **Peak**  **No.** | **Relative retention times (min)** | | | | | | **RSD**  **(%)** | **Relative peak areas** | | | | | | **RSD**  **(%)** |
| --- | --- | --- | --- | --- | --- | --- | --- | --- | --- | --- | --- | --- | --- | --- |
|  | **P_1_** | **P_2_** | **P_3_** | **P_4_** | **P_-5_** | **P_6_** |  | **P_1_** | **P_2_** | **P_3_** | **P_4_** | **P_-5_** | **P_6_** |  |
| 1 | 1.00 | 1.00 | 1.00 | 1.00 | 1.00 | 1.00 | 0.00 | 1.00 | 1.00 | 1.00 | 1.00 | 1.00 | 1.00 | 0.00 |
| 2 | 1.44 | 1.45 | 1.45 | 1.44 | 1.44 | 1.44 | 0.36 | 0.33 | 0.31 | 0.36 | 0.30 | 0.31 | 0.31 | 6.96 |
| 3 | 1.74 | 1.76 | 1.76 | 1.75 | 1.75 | 1.75 | 0.44 | 1.90 | 1.90 | 1.79 | 1.74 | 1.71 | 1.73 | 4.87 |
| 4 | 3.12 | 3.12 | 3.11 | 3.12 | 3.11 | 3.11 | 0.09 | 0.15 | 0.15 | 0.15 | 0.15 | 0.15 | 0.15 | 1.07 |
| 5 | 3.17 | 3.16 | 3.16 | 3.17 | 3.16 | 3.16 | 0.10 | 0.10 | 0.10 | 0.10 | 0.10 | 0.10 | 0.10 | 1.92 |
| 6 | 3.21 | 3.21 | 3.20 | 3.21 | 3.20 | 3.20 | 0.08 | 0.08 | 0.08 | 0.08 | 0.08 | 0.08 | 0.08 | 1.34 |
| 7 | 3.25 | 3.24 | 3.24 | 3.25 | 3.24 | 3.23 | 0.16 | 0.12 | 0.11 | 0.11 | 0.11 | 0.10 | 0.10 | 4.03 |
| 8 | 3.40 | 3.40 | 3.39 | 3.40 | 3.40 | 3.39 | 0.18 | 2.26 | 2.20 | 2.19 | 2.20 | 2.21 | 2.22 | 1.11 |
| 9 | 4.19 | 4.19 | 4.19 | 4.18 | 4.18 | 4.18 | 0.06 | 0.05 | 0.05 | 0.05 | 0.05 | 0.05 | 0.05 | 3.90 |
| 10 | 4.23 | 4.23 | 4.23 | 4.22 | 4.22 | 4.22 | 0.09 | 0.37 | 0.37 | 0.37 | 0.36 | 0.37 | 0.37 | 0.90 |
| 11 | 4.35 | 4.35 | 4.34 | 4.34 | 4.34 | 4.34 | 0.05 | 1.43 | 1.43 | 1.42 | 1.42 | 1.42 | 1.42 | 0.10 |
| 12 | 4.41 | 4.41 | 4.41 | 4.41 | 4.41 | 4.41 | 0.06 | 0.93 | 0.93 | 0.93 | 0.92 | 0.92 | 0.92 | 0.23 |
| 13 | 4.48 | 4.48 | 4.48 | 4.48 | 4.48 | 4.47 | 0.06 | 0.59 | 0.59 | 0.59 | 0.59 | 0.59 | 0.59 | 0.28 |
| 14 | 4.64 | 4.64 | 4.63 | 4.63 | 4.63 | 4.63 | 0.07 | 0.38 | 0.38 | 0.38 | 0.38 | 0.38 | 0.38 | 0.19 |
| 15 | 4.87 | 4.87 | 4.88 | 4.87 | 4.87 | 4.87 | 0.04 | 0.04 | 0.05 | 0.05 | 0.05 | 0.05 | 0.05 | 1.89 |
| 16 | 5.09 | 5.09 | 5.08 | 5.08 | 5.08 | 5.09 | 0.05 | 0.03 | 0.03 | 0.03 | 0.04 | 0.03 | 0.03 | 0.97 |
| 17 | 5.14 | 5.14 | 5.14 | 5.14 | 5.14 | 5.14 | 0.05 | 0.06 | 0.06 | 0.06 | 0.06 | 0.06 | 0.06 | 4.33 |
| 18 | 5.23 | 5.23 | 5.23 | 5.23 | 5.22 | 5.23 | 0.04 | 0.05 | 0.05 | 0.05 | 0.05 | 0.05 | 0.05 | 1.06 |
| 19 | 5.30 | 5.30 | 5.30 | 5.30 | 5.30 | 5.30 | 0.07 | 0.09 | 0.09 | 0.09 | 0.09 | 0.10 | 0.10 | 1.56 |
| 20 | 5.39 | 5.39 | 5.39 | 5.39 | 5.39 | 5.39 | 0.05 | 0.08 | 0.08 | 0.08 | 0.08 | 0.08 | 0.08 | 0.80 |
| 21 | 5.42 | 5.43 | 5.43 | 5.43 | 5.42 | 5.42 | 0.09 | 0.07 | 0.07 | 0.07 | 0.07 | 0.07 | 0.07 | 0.85 |
| 22 | 5.56 | 5.56 | 5.56 | 5.56 | 5.56 | 5.56 | 0.04 | 0.19 | 0.18 | 0.18 | 0.18 | 0.18 | 0.18 | 0.43 |
| 23 | 5.86 | 5.86 | 5.86 | 5.86 | 5.86 | 5.86 | 0.03 | 0.02 | 0.02 | 0.02 | 0.02 | 0.02 | 0.02 | 1.26 |
| 24 | 5.97 | 5.98 | 5.97 | 5.98 | 5.97 | 5.97 | 0.06 | 0.10 | 0.10 | 0.10 | 0.10 | 0.10 | 0.10 | 0.18 |
| 25 | 6.04 | 6.05 | 6.05 | 6.04 | 6.05 | 6.04 | 0.04 | 0.14 | 0.14 | 0.14 | 0.14 | 0.14 | 0.14 | 0.16 |
| 26 | 6.63 | 6.63 | 6.63 | 6.62 | 6.62 | 6.63 | 0.04 | 0.18 | 0.18 | 0.18 | 0.18 | 0.18 | 0.18 | 1.12 |
| 27 | 6.74 | 6.74 | 6.74 | 6.77 | 6.74 | 6.74 | 0.18 | 0.31 | 0.39 | 0.34 | 0.34 | 0.36 | 0.33 | 7.86 |
| 28 | 7.01 | 7.01 | 7.01 | 7.01 | 7.01 | 7.01 | 0.02 | 0.41 | 0.43 | 0.42 | 0.39 | 0.42 | 0.41 | 2.98 |

**Table S2.** Repeatability evaluation: relative retention times and relative peak areas

| **Peak**  **No.** | **Relative retention times (min)** | | | | | | **RSD**  **(%)** | **Relative peak areas** | | | | | | **RSD**  **(%)** |
| --- | --- | --- | --- | --- | --- | --- | --- | --- | --- | --- | --- | --- | --- | --- |
|  | **R_1_** | **R_2_** | **R_3_** | **R_4_** | **R_5_** | **R_6_** |  | **R_1_** | **R_2_** | **R_3_** | **R_4_** | **R_5_** | **R_6_** |  |
| 1 | 1.00 | 1.00 | 1.00 | 1.00 | 1.00 | 1.00 | 0.00 | 1.00 | 1.00 | 1.00 | 1.00 | 1.00 | 1.00 | 0.00 |
| 2 | 1.43 | 1.43 | 1.43 | 1.43 | 1.43 | 1.43 | 0.23 | 0.31 | 0.32 | 0.22 | 0.25 | 0.29 | 0.31 | 13.56 |
| 3 | 1.74 | 1.74 | 1.74 | 1.74 | 1.74 | 1.74 | 0.00 | 1.79 | 1.84 | 1.83 | 1.84 | 1.84 | 1.81 | 1.15 |
| 4 | 3.09 | 3.09 | 3.09 | 3.09 | 3.09 | 3.09 | 0.08 | 0.14 | 0.13 | 0.13 | 0.14 | 0.14 | 0.14 | 2.08 |
| 5 | 3.14 | 3.14 | 3.14 | 3.14 | 3.14 | 3.13 | 0.10 | 0.09 | 0.09 | 0.08 | 0.09 | 0.09 | 0.09 | 3.52 |
| 6 | 3.17 | 3.17 | 3.18 | 3.17 | 3.17 | 3.17 | 0.05 | 0.08 | 0.08 | 0.08 | 0.07 | 0.07 | 0.08 | 3.07 |
| 7 | 3.21 | 3.21 | 3.21 | 3.21 | 3.21 | 3.21 | 0.06 | 0.11 | 0.10 | 0.10 | 0.10 | 0.10 | 0.10 | 4.90 |
| 8 | 3.37 | 3.37 | 3.37 | 3.37 | 3.37 | 3.37 | 0.01 | 2.13 | 2.09 | 2.04 | 2.05 | 2.05 | 2.04 | 1.75 |
| 9 | 4.15 | 4.14 | 4.15 | 4.15 | 4.15 | 4.15 | 0.09 | 0.05 | 0.04 | 0.05 | 0.03 | 0.03 | 0.05 | 22.28 |
| 10 | 4.19 | 4.19 | 4.19 | 4.19 | 4.19 | 4.19 | 0.00 | 0.35 | 0.35 | 0.35 | 0.34 | 0.35 | 0.35 | 1.19 |
| 11 | 4.31 | 4.31 | 4.31 | 4.31 | 4.31 | 4.31 | 0.06 | 1.35 | 1.34 | 1.33 | 1.33 | 1.33 | 1.34 | 0.42 |
| 12 | 4.37 | 4.37 | 4.37 | 4.37 | 4.37 | 4.37 | 0.05 | 0.87 | 0.88 | 0.87 | 0.87 | 0.86 | 0.87 | 0.56 |
| 13 | 4.44 | 4.44 | 4.44 | 4.44 | 4.44 | 4.44 | 0.04 | 0.56 | 0.57 | 0.55 | 0.55 | 0.55 | 0.56 | 1.25 |
| 14 | 4.59 | 4.59 | 4.59 | 4.59 | 4.59 | 4.59 | 0.00 | 0.36 | 0.36 | 0.36 | 0.36 | 0.36 | 0.36 | 0.31 |
| 15 | 4.84 | 4.84 | 4.83 | 4.83 | 4.84 | 4.84 | 0.05 | 0.04 | 0.04 | 0.04 | 0.04 | 0.04 | 0.04 | 0.69 |
| 16 | 5.04 | 5.04 | 5.04 | 5.04 | 5.04 | 5.04 | 0.04 | 0.03 | 0.04 | 0.02 | 0.02 | 0.04 | 0.04 | 18.91 |
| 17 | 5.10 | 5.10 | 5.10 | 5.10 | 5.10 | 5.10 | 0.00 | 0.05 | 0.06 | 0.06 | 0.06 | 0.06 | 0.06 | 5.34 |
| 18 | 5.18 | 5.18 | 5.19 | 5.18 | 5.18 | 5.18 | 0.02 | 0.05 | 0.05 | 0.06 | 0.06 | 0.06 | 0.05 | 5.01 |
| 19 | 5.25 | 5.25 | 5.25 | 5.25 | 5.25 | 5.25 | 0.00 | 0.09 | 0.10 | 0.10 | 0.10 | 0.10 | 0.10 | 4.91 |
| 20 | 5.34 | 5.34 | 5.34 | 5.34 | 5.34 | 5.34 | 0.00 | 0.08 | 0.09 | 0.09 | 0.09 | 0.09 | 0.09 | 4.20 |
| 21 | 5.38 | 5.38 | 5.38 | 5.38 | 5.38 | 5.38 | 0.03 | 0.06 | 0.07 | 0.07 | 0.07 | 0.07 | 0.07 | 4.35 |
| 22 | 5.52 | 5.52 | 5.52 | 5.52 | 5.52 | 5.52 | 0.00 | 0.18 | 0.18 | 0.18 | 0.18 | 0.18 | 0.18 | 0.74 |
| 23 | 5.81 | 5.81 | 5.81 | 5.81 | 5.81 | 5.81 | 0.00 | 0.02 | 0.02 | 0.02 | 0.02 | 0.02 | 0.02 | 2.64 |
| 24 | 5.93 | 5.93 | 5.93 | 5.93 | 5.93 | 5.93 | 0.00 | 0.10 | 0.11 | 0.10 | 0.11 | 0.11 | 0.10 | 3.57 |
| 25 | 6.00 | 6.00 | 6.00 | 6.00 | 6.00 | 6.00 | 0.03 | 0.13 | 0.14 | 0.14 | 0.14 | 0.14 | 0.14 | 4.22 |
| 26 | 6.57 | 6.57 | 6.57 | 6.57 | 6.57 | 6.57 | 0.05 | 0.17 | 0.17 | 0.17 | 0.17 | 0.17 | 0.17 | 1.85 |
| 27 | 6.68 | 6.68 | 6.68 | 6.68 | 6.68 | 6.68 | 0.03 | 0.29 | 0.19 | 0.18 | 0.19 | 0.21 | 0.25 | 20.05 |
| 28 | 6.94 | 6.94 | 6.95 | 6.95 | 6.95 | 6.94 | 0.05 | 0.38 | 0.42 | 0.37 | 0.38 | 0.39 | 0.37 | 4.79 |

**Table S3.** Stability evaluation: relative retention times and relative peak areas

| **Peak**  **No.** | **Relative retention times (min)** | | | | | | | **RSD**  **(%)** | **Relative peak areas** | | | | | | | **RSD**  **(%)** |
| --- | --- | --- | --- | --- | --- | --- | --- | --- | --- | --- | --- | --- | --- | --- | --- | --- |
|  | **S_1_** | **S_2_** | **S_3_** | **S_4_** | **S_5_** | **S_6_** | **S_7_** |  | **S_1_** | **S_2_** | **S_3_** | **S_4_** | **S_5_** | **S_6_** | **S_7_** |  |
| 1 | 1.00 | 1.00 | 1.00 | 1.00 | 1.00 | 1.00 | 1.00 | 0.00 | 1.00 | 1.00 | 1.00 | 1.00 | 1.00 | 1.00 | 1.00 | 0.00 |
| 2 | 1.45 | 1.45 | 1.45 | 1.44 | 1.45 | 1.45 | 1.45 | 0.04 | 0.28 | 0.28 | 0.20 | 0.20 | 0.25 | 0.27 | 0.28 | 16.03 |
| 3 | 1.76 | 1.76 | 1.76 | 1.76 | 1.76 | 1.76 | 1.76 | 0.02 | 1.64 | 1.61 | 1.62 | 1.63 | 1.62 | 1.62 | 1.59 | 0.70 |
| 4 | 3.13 | 3.13 | 3.13 | 3.13 | 3.14 | 3.13 | 3.13 | 0.08 | 0.12 | 0.13 | 0.13 | 0.13 | 0.13 | 0.13 | 0.13 | 1.56 |
| 5 | 3.42 | 3.42 | 3.42 | 3.42 | 3.42 | 3.42 | 3.42 | 0.00 | 0.08 | 0.08 | 0.08 | 0.08 | 0.09 | 0.08 | 0.09 | 3.65 |
| 6 | 4.20 | 4.19 | 4.20 | 4.19 | 4.20 | 4.20 | 4.20 | 0.05 | 0.07 | 0.07 | 0.07 | 0.07 | 0.07 | 0.07 | 0.07 | 3.58 |
| 7 | 4.25 | 4.25 | 4.25 | 4.25 | 4.25 | 4.25 | 4.25 | 0.01 | 0.10 | 0.09 | 0.09 | 0.09 | 0.09 | 0.09 | 0.09 | 3.14 |
| 8 | 4.36 | 4.36 | 4.36 | 4.36 | 4.36 | 4.36 | 4.36 | 0.03 | 1.90 | 1.92 | 1.87 | 1.88 | 1.87 | 1.87 | 1.89 | 1.13 |
| 9 | 4.43 | 4.43 | 4.43 | 4.43 | 4.43 | 4.43 | 4.44 | 0.02 | 0.03 | 0.04 | 0.03 | 0.02 | 0.04 | 0.04 | 0.04 | 26.46 |
| 10 | 4.50 | 4.50 | 4.50 | 4.50 | 4.50 | 4.50 | 4.50 | 0.03 | 0.31 | 0.31 | 0.31 | 0.30 | 0.32 | 0.32 | 0.32 | 2.53 |
| 11 | 4.65 | 4.65 | 4.65 | 4.65 | 4.65 | 4.65 | 4.65 | 0.01 | 1.21 | 1.21 | 1.21 | 1.21 | 1.23 | 1.22 | 1.22 | 0.53 |
| 12 | 4.90 | 4.90 | 4.90 | 4.90 | 4.90 | 4.90 | 4.90 | 0.00 | 0.79 | 0.79 | 0.78 | 0.78 | 0.79 | 0.79 | 0.79 | 0.56 |
| 13 | 5.11 | 5.12 | 5.11 | 5.11 | 5.11 | 5.11 | 5.12 | 0.02 | 0.51 | 0.50 | 0.50 | 0.50 | 0.56 | 0.51 | 0.51 | 5.74 |
| 14 | 5.17 | 5.17 | 5.17 | 5.17 | 5.17 | 5.17 | 5.17 | 0.00 | 0.32 | 0.32 | 0.32 | 0.32 | 0.32 | 0.33 | 0.32 | 0.34 |
| 15 | 5.25 | 5.25 | 5.25 | 5.25 | 5.25 | 5.25 | 5.25 | 0.03 | 0.05 | 0.05 | 0.05 | 0.05 | 0.06 | 0.05 | 0.05 | 4.59 |
| 16 | 5.42 | 5.42 | 5.42 | 5.41 | 5.42 | 5.42 | 5.42 | 0.03 | 0.04 | 0.04 | 0.04 | 0.04 | 0.04 | 0.04 | 0.04 | 1.00 |
| 17 | 5.46 | 5.46 | 5.45 | 5.46 | 5.46 | 5.46 | 5.46 | 0.03 | 0.03 | 0.03 | 0.03 | 0.03 | 0.03 | 0.03 | 0.03 | 3.79 |
| 18 | 5.59 | 5.59 | 5.59 | 5.59 | 5.59 | 5.59 | 5.59 | 0.03 | 0.05 | 0.05 | 0.05 | 0.05 | 0.05 | 0.05 | 0.05 | 1.75 |
| 19 | 5.89 | 5.89 | 5.89 | 5.89 | 5.89 | 5.89 | 5.89 | 0.03 | 0.05 | 0.05 | 0.05 | 0.05 | 0.04 | 0.04 | 0.04 | 1.31 |
| 20 | 5.42 | 5.42 | 5.42 | 5.41 | 5.42 | 5.42 | 5.42 | 0.03 | 0.08 | 0.08 | 0.08 | 0.08 | 0.08 | 0.08 | 0.08 | 1.75 |
| 21 | 5.46 | 5.46 | 5.45 | 5.46 | 5.46 | 5.46 | 5.46 | 0.03 | 0.07 | 0.07 | 0.07 | 0.07 | 0.07 | 0.07 | 0.07 | 1.49 |
| 22 | 5.59 | 5.59 | 5.59 | 5.59 | 5.59 | 5.59 | 5.59 | 0.03 | 0.06 | 0.06 | 0.06 | 0.06 | 0.06 | 0.06 | 0.06 | 2.50 |
| 23 | 6.01 | 6.00 | 6.00 | 6.01 | 6.01 | 6.01 | 6.01 | 0.07 | 0.16 | 0.16 | 0.16 | 0.16 | 0.16 | 0.16 | 0.16 | 0.31 |
| 24 | 6.08 | 6.08 | 6.07 | 6.07 | 6.08 | 6.08 | 6.08 | 0.06 | 0.02 | 0.02 | 0.02 | 0.02 | 0.02 | 0.02 | 0.02 | 7.09 |
| 25 | 6.08 | 6.08 | 6.07 | 6.07 | 6.08 | 6.08 | 6.08 | 0.06 | 0.09 | 0.09 | 0.09 | 0.09 | 0.09 | 0.09 | 0.09 | 1.56 |
| 26 | 6.66 | 6.66 | 6.66 | 6.66 | 6.66 | 6.66 | 6.66 | 0.00 | 0.12 | 0.12 | 0.11 | 0.11 | 0.11 | 0.12 | 0.12 | 0.90 |
| 27 | 6.78 | 6.77 | 6.78 | 6.77 | 6.78 | 6.78 | 6.78 | 0.03 | 0.16 | 0.15 | 0.15 | 0.15 | 0.15 | 0.15 | 0.16 | 2.63 |
| 28 | 7.04 | 7.04 | 7.04 | 7.04 | 7.04 | 7.04 | 7.04 | 0.03 | 0.39 | 0.35 | 0.39 | 0.36 | 0.39 | 0.36 | 0.36 | 4.76 |
